# Primary Immunodeficiencies Associated With Early-Onset Inflammatory Bowel Disease in Southeast and East Asia

**DOI:** 10.3389/fimmu.2021.786538

**Published:** 2022-01-13

**Authors:** Yoji Sasahara, Takashi Uchida, Tasuku Suzuki, Daiki Abukawa

**Affiliations:** ^1^ Department of Pediatrics, Tohoku University Graduate School of Medicine, Sendai, Japan; ^2^ Department of General Pediatrics, Gastroenterology and Hepatology, Miyagi Children’s Hospital, Sendai, Japan

**Keywords:** early-onset inflammatory bowel diseases, primary immunodeficiency diseases, exome sequencing, allogeneic hematopoietic stem cell transplantation, IL-10RA deficiency, XIAP deficiency

## Abstract

**Background:**

Causes of early-onset inflammatory bowel disease (IBD) vary, and primary immunodeficiency diseases (PIDs) are associated with early-onset IBD as monogenic disorders.

**Aim:**

This review investigates the prevalence, clinical manifestation, genetic profile, and treatment of patients with early-onset IBD in Southeast and East Asia.

**Methods:**

A systemic review of articles reporting PID patients associated with early-onset IBD in Southeast and East Asia was conducted.

**Results:**

The prevalence of PID associated with IBD was higher than that reported in western nations, and the frequency of patients with bloody stools as an early symptom was relatively higher in monogenic diseases. A total 13 (12.0%) of 108 patients with early-onset IBD were diagnosed as PID by exome sequencing and targeted gene panel analysis in Japan, including four patients with *XIAP*, three with *IL10RA*, and two or one patient with other gene mutations. In addition, ten patients were reported as having IL-10 receptor alpha (IL-10RA) deficiency in China and Hong Kong. Allogeneic hematopoietic stem cell transplantation was performed in patients with X-linked inhibitor of apoptosis deficiency, IL-10RA deficiency, or other PID as a curative treatment, and the preferable outcome of reduced-intensity conditioning and complete resolution of IBD symptoms and dysbiosis were achieved.

**Conclusion:**

Comprehensive molecular diagnosis has been widely applied to screen for patients with PID-associated IBD in Southeast and East Asia. These results contributed to the awareness of monogenic PID in early-onset IBD patients and their differences in clinical manifestations and genetic profiles compared to the patients in western counties.

## Introduction

Inflammatory bowel disease (IBD) is caused by various factors, including genetic background, host-microbe interactions, dysbiosis, and environmental factors (1, 2). Recent comprehensive genome-wide studies revealed that some patients with IBD have disease-causing mutations or single-nucleotide polymorphisms that increase the risk of IBD in adults (3). Pediatric patients with early-onset IBD (EO-IBD) currently defined as clinical manifestations and/or being diagnosed under the age of 10 years old, including with very early-onset IBD (VEO-IBD) under the age of 6 years old, have distinct clinical features from those of adult patients with IBD, and some of the patients show an unclassified histology distinct from that of classical ulcerative colitis (UC) or Crohn’s disease (CD).

The worldwide VEO-IBD consortium, created in 2014, has contributed to understanding the molecular basis of VEO-IBD and to the development of personalized treatments for patients with these rare diseases (4). It has been reported that genes responsible for primary immunodeficiency diseases (PID), including interleukin-10 (IL-10) or IL-10 receptor (IL-10R) deficiency, X-linked inhibitor of apoptosis (XIAP) deficiency, immune dysregulation, polyendocrinopathy, enteropathy, X-linked (IPEX) syndrome, Wiskott–Aldrich syndrome (WAS), chronic granulomatous disease (CGD), and common variable immunodeficiency, are involved in the molecular pathogenesis of pediatric IBD (5–7). Diagnostic approaches using exome sequencing and targeted gene panel analysis have contributed to the definite molecular diagnosis of early-onset IBD (8–12). Crowley et al. (2020) reported the prevalence and clinical symptoms of early-onset IBD patients enrolled from worldwide countries. They identified 40 rare variants associated with 21 disease-causing genes in 31 (3.1%) of 1,005 patients with IBD. These variants occurred in 7.8% of IBD patients younger than 6 years old and in 2.3% of children aged 6–18 years old. Of the 17 patients with monogenic CD, 35% experienced abdominal pain, 24% had non-bloody loose stool, 18% had vomiting, 18% had weight loss, and 5% had intermittent bloody loose stool. Of the 14 patients with monogenic UC or unclassified histology, their most predominant feature was bloody loose stool (78%). Twenty-two patients (2.2%) had variants in genes responsible for PID, including five variants in *XIAP;* three in *DOCK8;* two each in *FOXP3*, *LRBA* and *ARPC1B;* and one each in *IL10RB*, *CYBB*, and other genes. Only 1% of the patients with variants were considered potential candidates for correction of variants by allogeneic hematopoietic stem cell transplantation (HSCT) (13).

In accordance with the worldwide recognition, multicenter studies for pediatric patients with IBD have been reported in Japan, China, Hong Kong, and Malaysia in recent years. This review of relevant published literatures in the Southeast and East Asia summarizes the prevalence, clinical manifestations, results of genetic analysis by exome sequencing and targeted gene panel analysis, and treatment options of allogeneic HSCT as a curative therapy focusing on IL-10RA deficiency, XIAP deficiency and other PID.

## Methods

A comprehensive search of articles reporting PID patients associated with early-onset IBD in Southeast and East Asia was performed using PubMed (http://pubmed.ncbi.nlm.gov). The following search terms or abbreviations were used: primary immunodeficiency, PID, inflammatory bowel disease, IBD, early-onset IBD, VEO-IBD, Asia.

The present study was approved by the Ethics Committee of the Tohoku University Graduate School of Medicine.

## Prevalence and Clinical Manifestations

Lee et al. (2016) described the prevalence and clinical features of VEO-IBD in University Malaya Medical Center, Malaysia. Six patients (13%, CD = 3, UC = 2, IBD-unclassified = 1) out of 48 pediatric patients (CD = 25, UC = 23) of IBD were infantile-onset IBD before 12 months of age. Compared with later-onset IBD patients, infantile-onset IBD patients were more likely to present with bloody diarrhea; however, no mutation in IL-10 or IL-10R was identified in enrolled patients (14). Ishige et al. (2010) analyzed a national IBD registry database of Japanese patients treated between 2003 and 2006, and reported that 10.6% of CD patients and 5.9% of UC patients were under the age of 16 years old. They showed that pediatric patients with IBD had clinical features that are distinct from those in adult patients with IBD. In comparison with adults, pediatric patients more commonly had a positive family history of CD and UC, tended to have more severe disease, and more often had extensive colitis in UC (15). Maisawa et al. (16) retrospectively investigated the clinical features of children who were diagnosed with IBD between 1998 and 2008 in Japan, especially those whose onset were younger than 8 years of age. Totally, 24 patients with CD and 47 patients with UC were analyzed on the basis of the final diagnosis. Among patients with CD, the age at onset was less than 1 year in 62.5% of patients; 87.5% of CD patients involved the colon; and 63.8% of UC cases were pancolitis. Growth failure was more severe at diagnosis in CD patients than in UC patients. Familial occurrence within first-degree relatives was observed in eight families among 45 patients with UC, compared with none among CD patients. They indicated that the prevalence and clinical manifestations of IBD in infants and children in Japan differed from that in western countries in terms of earlier age at disease onset of CD and higher incidence of familial occurrence of UC (16). Suzuki et al. (17) analyzed 35 Japanese patients under 16 years of age who were suffering from severe and refractory IBD and were enrolled in this multicenter study, including 27 patients with VEO-IBD under 6 years of age. In total, 5 of 35 patients (14.3%), including 4 patients with VEO-IBD, were diagnosed with monogenic disorders (17). Subsequently, Uchida et al. (18), in the same institute analyzed 108 enrolled patients under 17 years of age who were suffering from early-onset diarrhea and were refractory to conventional therapies in Japan. They reported that a family history of Bechet’s disease was predominantly related to monogenic disease and that the frequency of patients with bloody stools as an early symptom was relatively higher in monogenic disease than in non-monogenic cases (18).

## Genetic Profiles

**Figure 1:**
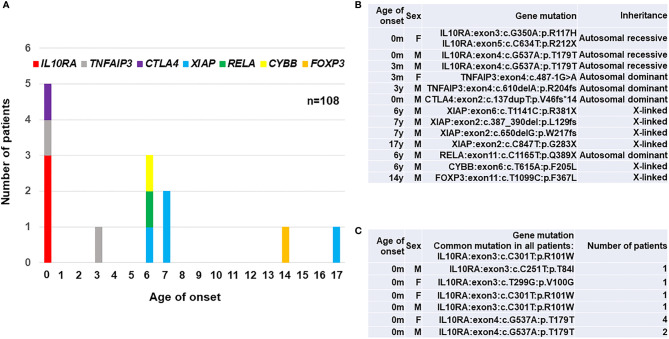
A summary of 23 patients with PID-associated early-onset IBD in East Asia. **(A)** Age distribution of disease onset according to the responsible genes in 13 Japanese patients. **(B)** A list of disease-causing mutations in responsible genes for PID in 13 Japanese patients (17, 18). **(C)** A list of disease-causing mutations in 10 patients with IL-10RA deficiency reported from Hong Kong (19) and China (20). A synonymous p.T179T variant caused a splicing error in *IL10RA* gene. IL10RA, interleukin-10 receptor subunit alpha; TNFAIP3, tumor necrosis factor alpha-induced protein 3; CTLA4, cytotoxic T-lymphocyte-associated protein 4; XIAP, X-linked inhibitor of apoptosis protein; RELA, v-rel avian reticuloendotheliosis viral oncogene homolog A; CYBB, cytochrome b beta; FOXP3, forkhead boxprotein P3; M, male; F, female.

We established exome sequencing and targeted gene panels covering all responsible genes for PID and early-onset diarrhea. The data revealed that a total of 15 (13.9%) out of 108 patients enrolled in the study were monogenic. A total of 13 (12.0%) patients were diagnosed as monogenic PID in Japan (17, 18), and the frequency of monogenic PID among early-onset IBD patients was relatively higher than that of 2.2% among 1,008 early-onset IBD patients reported in western counties (13). The different incidence of monogenic IBD between western countries and Japan may be caused by the criteria for enrolled patients, genetic background and other environmental factors. The age distribution of 13 monogenic PID patients in Japan is shown in **(Figure 1A)** and their actual disease-causing mutations in responsible genes for PID are shown in **(Figure 1B)**. The number of patients with gene mutations were four patients with *XIAP*, three with *IL10RA*, two with *TNFAIP3*, and one each with *RELA*, *CTLA4*, *FOXP3*, and *CYBB* gene mutations. All three patients with IL-10RA deficiency were under 1 year of age; all four patients with XIAP deficiency were over 6 years of age at onset. The patient with refractory diarrhea caused by heterozygous truncated RelA protein expression was the first identified case worldwide, and functional analysis revealed that the mutation affected nuclear factor-kappa b (NFκB) signaling. Genotypes were significantly associated with clinical and pathological findings in each patient. Yanagi et al. (21) and Ishige et al. (22) reported another patient with IL-10RA deficiency. Ishihara et al. (23) reported a rare patient with Hermansky–Pudlak syndrome in Japan (23).

Ten patients with IL-10RA deficiency were reported in Hong Kong and China as listed in **Figure 1C**. Mao et al. (2012) in Hong Kong identified a patient with compound heterozygous mutations in the *IL10RA* gene in 2012 (24). Peng et al. (2018) in China diagnosed up to nine patients with IL-10RA deficiency by exome sequencing and treated them using reduced-intensity conditioning (RIC) followed by umbilical cord blood transplantation (CBT) (19). All ten patients showed initial manifestations within one month after birth and had a common mutation of c.C301T (p.R101W) in *IL10RA* gene. These findings indicate that the frequency of IL-10RA deficiency in China is higher than in western countries. Moreover, predictive prenatal diagnosis for IL-10RA deficiency in eight families was performed in China, although the legal and ethical considerations are still controversial for prenatal diagnoses of diseases for which curative treatments are available after birth (24).

Kammermeier et al. (2014) and Uhlig et al. (2021) proposed integrated diagnostic methods and clinical genomics for IBD-related PID patients based on their different implications in systemic immunity (11, 12). Genetic profiles in most of IBD-related PID patients in East Asia can be sorted into the algorithms, although molecular pathogenesis of a patient with a heterogenous *RELA* mutation remained to be clarified.

## Treatments

Genetic investigations provided us a new approach for better treatments other than immunosuppressive agents, nutritional supplement and surgical intervention. One of the curative treatments for PID with early-onset IBD is allogeneic HSCT, and RIC is a suitable treatment option for these nonmalignant diseases in terms of reduced long-term sequelae if engraftment and complete chimera are achieved after HSCT. Allogeneic HSCT can induce remission in patients with IL-10 and/or IL-10R deficiency (25, 26). In Japan, only two patients with IL-10RA deficiency were reportedly treated successfully with RIC followed by allogeneic HSCT and achieved disease remission after engraftment (21, 27). In China, nine patients with severe clinical conditions with IL-10RA deficiency received RIC followed by allogeneic CBT, and six patients achieved complete remission without evidence of graft-vs.-host disease (GVHD) or infections. However, one patient died of chronic lung GVHD at 6 months post-transplantation, and the other two patients died of sepsis due to unsuccessful engraftments. Severe malnutrition and growth retardation associated with the disease were significantly improved in engrafted patients (19). In addition, Neven et al. (2013) reported a Mendelian predisposition to B-cell lymphoma caused by IL-10R deficiency, and allogeneic HSCT was a curative treatment for the life-threatening complication (28). Therefore, optimal RIC regimens for successful engraftment should be discussed in IL-10RA deficiency.

**Figure 2:**
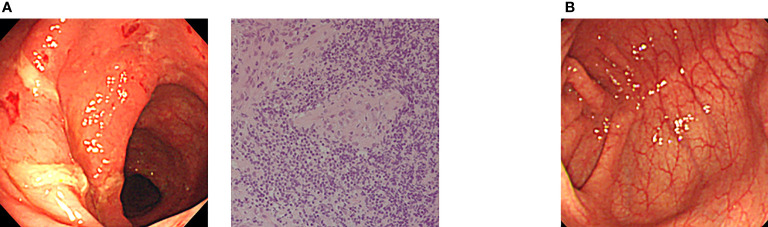
Amelioration of endoscopic findings after allogeneic CBT in a Japanese patient with XIAP deficiency. **(A)** Endoscopic evaluation at onset showed edema, hemorrhage and ulcer formation. Pathological evaluation of biopsy specimen at onset showed nonspecific active inflammation. **(B)** Completely ameliorated endoscopic finding after allogeneic CBT in the same patient (17, 33).

Allogeneic HSCT has also been applied for IBD in XIAP deficiency as a curative treatment option (29–31). Yang et al. (2012) reported clinical and genetic characteristics of XIAP deficiency in Japan, and established flowcytometric analysis of XIAP protein expression in lymphocytes, which was useful for determining engraftment and chimeric status in each hematopoietic cell lineage (32). Ono et al. (2017) reported a nationwide survey of details of allogeneic HSCT for XIAP deficiency in Japan. They showed that the optimal RIC regimen containing hemophagocytic lymphohistiocytosis control by etoposide and dexamethasone palmitate might be important factors for successful outcomes and improved IBD symptoms in patients with XIAP deficiency. Nine of the 10 patients were alive and well at a median of 21.2 months after HSCT (33). They recently reported that IBD associated with XIAP deficiency is caused by dysbiosis of the gut microbiota, and allogeneic HSCT ameliorated gut inflammation and dysbiosis in patients with XIAP deficiency (34). Amelioration of IBD symptoms is expected to improve the quality of life of patients treated with HSCT. Our endoscopic and histological findings revealed that colon symptoms significantly ameliorated after HSCT in Japanese patients with XIAP deficiency (**Figure 2**).

Other PID-associated IBDs included IPEX syndrome caused by *FOXP3* gene mutation and the absence of regulatory T cells (Tregs). Horino et al. (2014) reported a selective expansion of donor-derived Tregs after allogeneic bone marrow transplantation in a mixed chimeric state in a patient with IPEX syndrome, thus suggesting a growth advantage of normal Tregs in the IPEX patient. He suffered from severe diarrhea and required parenteral nutrition and red blood cell transfusion because of severe bloody diarrhea. The gastrointestinal symptoms were ameliorated after engraftment and expansion of CD4+CD25+Foxp3+ Tregs. However, optimal conditioning regimens should be further discussed to achieve a complete chimeric state in the rare patients with IPEX syndrome (35).

If the patients had no human leukocyte antigen (HLA)-identical donors or suitable cord blood for transplantation, an HLA haplo-identical related donor was another candidate for HSC sources. Osumi, et al. (2020) reported a retrospective study of allogeneic HSCT with post-transplantation cyclophosphamide and antithymocyte globulin from HLA-mismatched related donors for nonmalignant diseases, and XIAP deficiency, IL-10RA deficiency, WAS and CGD patients were included in the case series (27).

In terms of treatments other than HSCT, the frequency of patients treated with gastrointestinal surgery was relatively higher in the monogenic patients than in non-monogenic IBD patients (15–18). The relationship between the usage rate of prednisolone (PSL) or anti-TNF-α antibody and the monogenic disease was not clear in Japan. PSL was effective for 55.1% of non-monogenic and 40.0% of monogenic IBD patients. The anti-TNF-α antibody was effective for 69.4% of non-monogenic and 37.5% of monogenic IBD patients, suggesting that the refractory properties of the anti-TNF-α antibody were conspicuous in monogenic patients. As one of other therapeutic options, the effective treatments with an abatacept, a fusion protein of the extracellular domain of the human cytotoxic T-lymphocyte-associated protein 4 (CTLA4) linked to a modified Fc of human IgG1, were reported in patients with monogenic IBD caused by lipopolysaccharide (LPS)-responsive and beige-like anchor (LRBA) deficiency and cytotoxic T-lymphocyte-associated protein 4 (CTLA4) deficiency in China (36) and worldwide (37).

## Implication for Immunity in Gastrointestinal Tract

The genes responsible for PID play critical roles in normal immunity in the gastrointestinal tract. Using whole-exome sequencing and targeted gene panel analysis, they identified underlying gene mutations responsible for PID in pediatric patients with early-onset IBD in an East Asian population. We are aware of the evidence that normal IL-10 signaling, nucleotide-binding oligomerization domain-containing protein 2 (NOD2)-mediated signaling and Tregs play indispensable roles in keeping normal immune homeostasis in the gastrointestinal tract by determining immunological defects in patients with IL-10 signaling deficiency, XIAP deficiency and IPEX syndrome, respectively.

Classical WAS patients also suffer from VEO-IBD (38, 39). A complex of Wiskott-Aldrich syndrome protein (WASP) and WASP-interacting protein (WIP) is recruited to the immunological synapse between antigen presenting cells and T cells, regulating T cell receptor-driven IL-2 production and actin polymerization in T cells (40–42). A fraction of WASP is localized in the nucleus and loss of WASP induces impaired T helper 1 (Th1) cell functions and Th2-dominant immunity (43). Nguyen et al. (2007) reported that a relative Th2 cytokine predominance is critical for the colitis in WASP-deficient mice (44). They also reported that defective interactions between WASP-deficient innate immune cells and T cells induced the dysfunctions of tolerogenic dendritic cells, impaired IL-10 signaling and homeostasis of Tregs in mouse model (45). Therefore, Th2-colitis, defective IL-10 signaling and Tregs are involved in the pathogenesis of colitis in WAS patients.

We recently reported that a pig model of X-lined severe combined immunodeficiency (X-SCID) completely lacked Peyer’s patches and IgA production in the small intestine. Allogeneic HSCT to X-SCID pigs did not facilitate the lymphoid organogenesis completely and created atypical intestinal immune and microbial environments in the animal model of X-SCID. We also showed that our patients with X-SCID in mixed chimera after current allogeneic HSCT showed lower IgA levels and dysbiosis in their stool samples compared to the patients in complete chimera or normal individuals, indicating that common γ chain had significant roles in intestinal lymphoid organogenesis (46).

## Conclusion

Comprehensive molecular diagnosis has been widely applied to screen monogenic PID with early-onset IBD patients in Southeast and East Asia. The findings of different studies and contrasting issues compared to western counties are relatively higher frequency of IBD patients associated with various PID in Japan and higher frequency of IL-10RA deficiency in China and Hong Kong. Identifying links between genetic mutations and clinicopathological and immunological parameters helped us understand the pathogenesis and select appropriate therapies, such as infliximab, immunosuppressive therapy, or allogeneic HSCT as a curative treatment for patients with IBD. Determining whether unidentified genes, a dysregulated immune response to intestinal microbiota, or dysbiosis are involved in the pathogenesis of these diseases may expand our understanding of normal immunity and host-microbe interactions in the gastrointestinal tract.

## Author Contributions

All authors listed have made a substantial, direct, and intellectual contribution to this work and approved it for publication.

## Funding

This work was supported by grants from the Japanese Ministry of Health, Labour and Welfare (20FC1047, 20FC1053); the Japan Agency for Medical Research and Development (JP21gk01104h0003); and the Japan Blood Products Organization to YS.

## Conflict of Interest

The authors declare that the research was conducted in the absence of any commercial or financial relationships that could be construed as a potential conflict of interest.

The handling editor declared a past collaboration with the authors YS, TU, and TS.

## Publisher’s Note

All claims expressed in this article are solely those of the authors and do not necessarily represent those of their affiliated organizations, or those of the publisher, the editors and the reviewers. Any product that may be evaluated in this article, or claim that may be made by its manufacturer, is not guaranteed or endorsed by the publisher.
